# Delving into the Causes and Effects of Entomopathogenic Endophytic *Metarhizium brunneum* Foliar Application-Related Mortality in *Spodoptera littoralis* Larvae

**DOI:** 10.3390/insects11070429

**Published:** 2020-07-09

**Authors:** Inmaculada Garrido-Jurado, David Montes-Moreno, Pilar Sanz-Barrionuevo, Enrique Quesada-Moraga

**Affiliations:** Department of Agronomy, ETSIAM, University of Cordoba, Campus de Rabanales, Edificio C4 Celestino Mutis, 14071 Cordoba, Spain; g32momod@uco.es (D.M.-M.); g32sabam@uco.es (P.S.-B.); cr2qumoe@uco.es (E.Q.-M.)

**Keywords:** ascomycete, mycoinsecticide, cotton leafworm, fitness, caspase, tritrophic interaction

## Abstract

The aim of the current study was to delve into the causes of mortality of *Spodoptera littoralis* larvae feeding on *Metarhizium*-colonized plants in the absence of fungal outgrowth on the cadavers as previous studies reported and to elucidate the possible indirect effects of this fungus-colonized diet. The effect was evaluated in experiments conducted using leaf discs of colonized plants and in planta using fungus-colonized whole plants. The mortality rates of larvae fed on *Metarhizium*-colonized melon leaves were 45.0% and 87.5%, and the average survival times were 6.6 and 3.1 days in experiments performed with discs and *in planta,* respectively. Notably, these mortality levels were not associated with observed apoptosis mediated by caspases 1, 3-7 and 8; thus, further investigation into the possible immune system reaction of the insect after the ingestion of colonized plants is required. The leaf consumption of *S. littoralis* larvae fed on melon-colonized leaves was lower than that on control plants in the disc experiments but not in experiments conducted *in planta*. In this regard, in experiments performed *in planta*, plant damage increased larval mortality in both fungally challenged and control larvae. There was also a meaningful effect of exposure to *Metarhizium*-colonized melon leaf discs on *S. littoralis* fitness, with significant reductions in 39.0% and 22.0% in female fecundity and egg fertility, respectively, detected in females emerging from pupae developing from larvae surviving exposure to colonized plant discs; all larvae died in the *in planta* experiments. Hence, the present work presents new findings revealing the high potential of endophytic entomopathogenic fungi to improve the outcome of foliar applications against chewing insects in the short, mid- and long term, by the reduction of the reproductive potential of surviving adults and reveals new insights into the development of bioassays with whole plants for more detailed evaluation of the impact of these fungi as endophytes used for plant protection.

## 1. Introduction

The use of entomopathogenic fungi (EPFs) in integrated pest management programs as an alternative to less environmentally benign alternatives, such as chemical insecticides, is becoming more frequent [1]. EPFs are contact action microbials that are normally applied via spray application directed at the plant canopy targeting a pest; depending upon the endophytic behavior of the fungal strain, this may lead to temporary colonization of the leaves that improves the outcome of foliar applications of mycoinsecticides against piercing–sucking and chewing insects [2,3]. Strategies such as soil treatments and seed dressing, can also take advantage of EPF endophytic behavior, potentially leading to the systemic protection of the plant against insect and mite pests [1,4]. In this regard, both temporary and permanent endophytic colonization has provided successful control of boring, chewing, and sucking insects [1,2,3,5,6].

The polyphagous cotton leafworm, *Spodoptera littoralis* (Boisduval) (Lepidoptera: Noctuidae), is distributed worldwide and can feed on more than 100 host plants, potentially producing yield losses of 50% related to its larval foliage consumption activity [7,8,9]. Its control is becoming more difficult due to resistance and cross-resistance to chemical insecticides and to the bioinsecticide *Bacillus thuringiensis* Berliner [10,11]. However, the use of EPFs and the insecticidal compounds they produce has shown promise against this harmful lepidopteran in both foliar- or soil-dwelling stages and exhibits compatibility with nontarget organisms, including natural enemies [3,12,13,14,15,16,17]. To date, there are many studies that report the success of using different genera of EPF (*Metarhizium anisopliae* (Metsch.), *Beauveria bassiana* (Balsamo) Vuill., *Cordyceps fumosorosea* (Wize) Kepler, B. Shrestha & Spatafora among others) for controlling different stages of *S. littoralis* [18,19,20], but only a few have focused on the new role of EPF as an endophyte for control of this pest [3,7,17].

Notably, *S. littoralis* larvae and larvae from other lepidopterans that die after being fed plants endophytically colonized with EPFs rarely show any signs of fungal outgrowth, with the possible causes of mortality and effects on pest fitness remaining unknown [3,17,21]. Indeed, recent studies addressing EPF-arthropod interactions have reported that the EPFs may induce arthropod death without the classical fungal infection process involving conidial adhesion, germination and penetration through the cuticle. For example, the ingestion of *Metarhizium* sp. conidia by mosquito larvae and the aquatic crustaceans *Daphnia pulex* and *Artemia magna* induces high levels of stress gene expression and stress-mediated caspase activation, which are the cause of their death, rather than fungal colonization [22,23,24]. Similarly, the mechanical damage caused to the plant when obtaining leaf discs for experiments may result in the loss of volatiles and production of secondary metabolites and enzymes that modify the fitness and behavior of insects [25,26], which could occur in bioassays in which EPF-colonized leaf discs are offered to lepidopterans [25]. The aim of the present work was to explore the effects of feeding *S. littoralis* larvae on *Metarhizium*-colonized melon leaves on their mortality and leaf consumption and the fecundity and fertility of the adults developing from surviving larvae. In addition, this work examines the possible causes of *S. littoralis* mortality after feeding on *Metarhizium*-colonized plants, with an emphasis on plant defense- and host defense-related mechanisms such as apoptosis.

## 2. Materials and Methods

### 2.1. Insects

*Spodopera littoralis* larvae were obtained from a stock colony from the Department of Agronomy of the University of Cordoba (Spain). The larvae were maintained at 26 ± 2 °C under 70 ± 5% RH and a photoperiod of 16:8 (L:D) h, and they were fed an artificial diet as described by Santiago-Álvarez [27].

### 2.2. Fungal Strain

*Metarhizium brunneum* strain EAMb 09/01-Su was obtained from the culture collection at the Department of Agronomy of the University of Cordoba (Spain). This strain was originally isolated from the soil of a meadow forest system in Castilblanco de los Arroyos (Seville, Spain). The strain was deposited in the Spanish collection of culture types (CECT) with accession number CECT 20784. Cultivation and inoculum production for the laboratory and field experiments were performed as described by Garrido-Jurado et al. [2]. This strain was selected due to both its high virulence towards *S. littoralis* and its endophytic behavior [3].

### 2.3. Colonization of Melon Plants

Melon seeds (galia variety) were disinfected by immersion in 70% (*v*/*v*) ethanol for 2 min, followed by 2% NaOCl (Sigma-Aldrich, St. Louis, MO, USA) for 5 min. Then, they were transferred to plastic pots with 110 wells (25 × 25 × 25 mm) containing the sterilized substrate “Compo Sana plantation” (Compo GmbH, Münster, Germany). One seed was sown per pot, and the pots were maintained at a temperature of 27 ± 3 °C under 14 h of daylight (light intensity of 700 lx) and 10 h of darkness. The plants were subsequently transplanted to individual pots (70 × 70 × 60 mm) containing 300 g of sterilized substrate and maintained at 26 ± 5 °C with a 14:10 h photoperiod.

**Figure 1:**
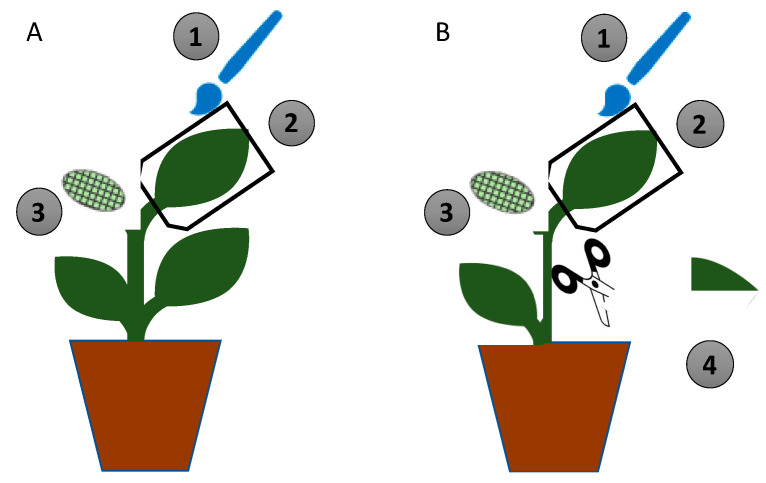
Design of the in-planta experiment. (**A**) Non-damaged plant; (**B**) damaged plant. (1) One leaf was brushed with the fungal suspension (treated) or sterile water with 0.1% Tween 80 (control). (2) The leaf was covered with a transparent plastic bag for 48 h. (3) Four L_2_ *Spodoptera littoralis* larvae were clipped on a non-treated leaf at 48 h and left to feed on the leaf for 48 h. (4) Damage was artificially caused by tearing a non-treated leaf at 48 h after fungal inoculation.

For experiments with discs, a fungal suspension of 10^8^ conidia mL^−1^ with 0.1% Tween 80 was sprayed on 6 true-leaf plants with an aerograph 27085 (piston compressor of 23 L/min, 15-50 PSI and 0.3 mm nozzle diameter, Artesanía Latina S.A., Lamadrid, Spain). The leaves were sprayed with 2 mL of the fungal suspension, and the soil was covered with aluminum foil to avoid the run-off of the suspension. Thereafter, the plants were covered with a transparent plastic sheet to promote fungal growth for 48 h. Control plants were sprayed with sterile water with 0.1% Tween 80. For *in-planta* experiment, one leaf per plant was brushed with a fungal suspension (10^8^ conidia mL^−1^ with 0.1% Tween 80), and the control was brushed with sterile water with 0.1% Tween 80. The leaves were then covered with a transparent plastic bag to promote fungal growth for 48 h (Figure 1).

The leaves of plants colonized for 48 h and controls were removed from the plants and washed with a 2% aqueous solution of NaOCl for 5 min, followed by two rinses of sterilized water for surface disinfection. To ensure total disinfection of the leaves, 100 µL of the last rinse from each sample was cultured on malt agar (MA) plates. For the in-planta experiment, one non-treated leaf from treated and control plants was also inspected for endophytic colonization following the previous protocol. Subsequently, ten fragments per leaf (5 × 5 mm) were randomly collected and placed on MA plates, ensuring full contact between fragments, and culture was performed in medium at 25 °C. Colonization was determined by counting the number of fragments showing fungal outgrowth after 7 days of incubation. To confirm the fungal infection of the fragments, the fungus was observed using a light microscope (Nikon, Tokyo, Japan) and identify its morphological features according to Seifert et al. [28].

### 2.4. Effect of Exposure of S. littoralis to Metarhizium-Colonized Melon Discs

Third-instar larvae of *S. littoralis* were individually fed 10-mm colonized melon discs, while the controls were fed melon discs from control plants. The discs were previously surface-disinfected using the procedure described in Section 2.3. The discs were replaced by other fresh discs daily and then scanned to measure the amount of consumed foliar leaf. Three replicates of 10 larvae each were used per treatment, and the treatments were repeated four times. First, the direct effect on larval mortality was recorded every 24 h for 8 days, as was the consumed area of the melon discs. In addition, the non-direct effects that were measured included (1) apoptosis in the dead larvae based on the measurement of caspase activity using Caspase Glo 1 Inflammasome, Caspase Glo 3-7, and Caspase Glo 8 luminometric kits in accordance with the manufacturer’s guidelines (Promega), as described by Garrido-Jurado et al. [24], and (2) the development of living larvae, followed until their pupation and the emergence of adults. As adults emerged, they were placed in oviposition chambers, and egg clusters were collected as described by Garrido-Jurado et al. [15]. The total numbers of eggs laid per female and newly emerged larvae were recorded.

### 2.5. Effect of the in Planta Exposure of S. littoralis to Metarhizium-Colonized Melon Leaves

One non-treated leaf of each plant that had been treated for 48 h-treated was infested with four L_2_ *S. littoralis* larvae using clip cages (Figure 1). The clip cages were removed 48 h post-infestation, and the larvae were placed in individual cages, as described in Section 2.4, after which they received an artificial diet. Similarly, control plants were infested with four L_2_ *S. littoralis* larvae. Other 48-h-treated plants were also infested as described above, but damage was artificially caused by tearing a non-treated leaf. The last procedure was also performed in the control plants. As in the previous treatments, clip cages were removed 48 h post-infestation, and the larvae were reared individually and fed an artificial diet thereafter. There were three plants per treatment and the experiment was performed twice. The leaves infested with larvae were scanned to measure the consumed area. The treated leaves and consumed leaves were surface disinfected using the procedure described in Section 2.3 and placed in MA plates to evaluate the colonization rate. Larval mortality was recorded every 24 h for 8 days, and the Caspase Glo 1 Inflammasome assay was followed, as in Section 2.4, since only caspase 1 is specific to lepidopterans [29].

### 2.6. Statistical Analysis

Mortality data and female fertility data were analyzed using a generalized linear model (distribution = binomial; link = logit). Female fecundity data were also modelled with a generalized linear model (distribution = Poisson; link = log) using JMP 8 software (SAS^®^, Cary, NC, USA). Treatment comparisons were performed using the χ^2^ test (*p* < 0.05). Average survival times (ASTs) and cumulative survival ratios were obtained via Kaplan–Meier survivorship analysis and compared via the log-rank test calculated with IBM SPSS 25.0 software. Digital images of the melon discs and leaves were captured to evaluate the leaf consumption of each larva. The area of each disc/leaf was delimited using ImageJ (http://imagej.nih.gov/ij/), and the consumed area of melon discs was analyzed by analysis of variance (ANOVA) and compared using the LSD test at 5% significance since the discs were replaced daily, following the confirmation of normality and homogeneity of variance for the raw data. The consumed areas of the leaves in the in-planta experiment were analyzed according to Kruskal–Wallis one-way nonparametric AOV analysis. All analyses of the consumed area were performed using IBM SPSS 25.0 software.

## 3. Results

### 3.1. Effect of Exposure of S. littoralis to Metarhizium-Colonized Melon Discs

#### 3.1.1. Mortality and Food Consumption

**Figure 2:**
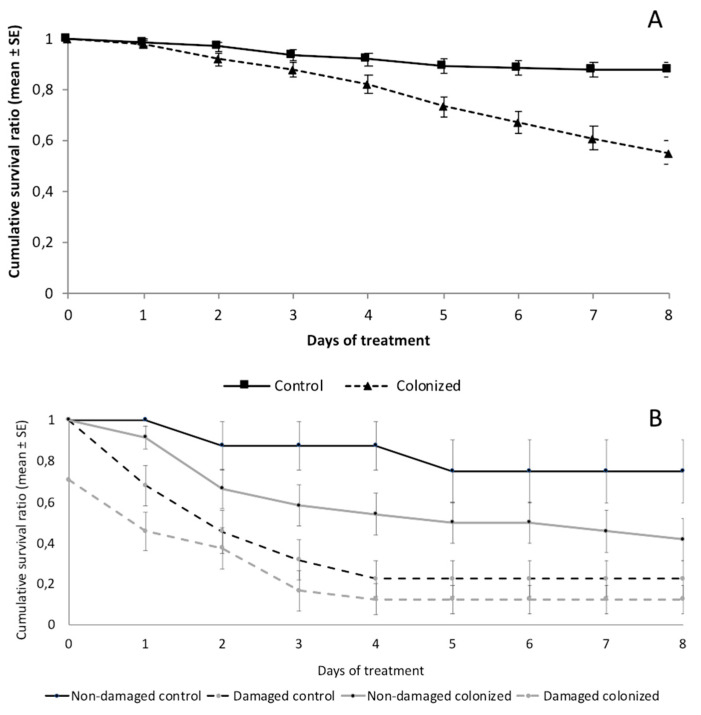
Cumulative survival ratio (mean ± SE) of *S. littoralis* larvae fed on (**A**) control (n = 120) and *Metarhizium*-colonized melon discs (n = 120), (**B**) control and *Metarhizium*-colonized plants in experiments with whole plants (non-damaged) (n_control_ = 24, n_colonized_ = 24) and plants damaged by tearing a non-treated leaf (damaged) (n_control_ = 24, n_colonized_ = 24).

The percentage of the fungal colonization of the treated melon leaves was 64.4 ± 6.8% at 48 h, whereas fungal colonization was not detected in the control leaves. Significant differences in mortality [χ^2^(1) = 34.09, *p* < 0.001] were found between larvae fed on the control and *Metarhizium*-colonized discs, with mortality rates of 11.7 and 45.0%, respectively, and Abbott mortality of 33.3% (Table 1). The average survival times also showed significant differences and were 6.6 days for the fungal treatment and 7.5 days for the controls (Table 1). In addition, the cumulative survival ratio significantly decreased in the larvae fed melon leaves colonized by the fungus (Figure 2A).

**Figure 3:**
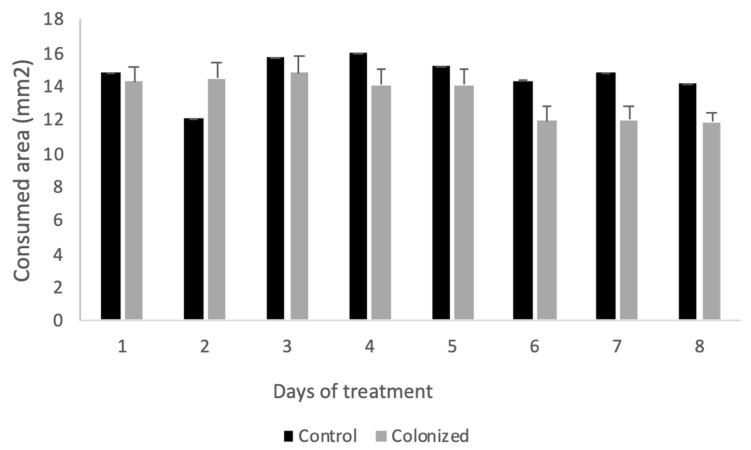
Mean area (mm^2^) of control and *Metarhizium*-colonized discs consumed by *S. littoralis* larvae during the experiment (8 days). Bars indicate ± SE. n_control_ = 720, n_colonized_ = 720.

There were significant treatment (F_1,1192_ = 238.8, *p* = 0.0011), time-related (F_7,1192_ = 1065.0, *p* < 0.0001) and treatment–time interaction (F_7,1192_ = 448.6, *p* < 0.0001) differences in the mean area (mm^2^) of the control and *Metarhizium*-colonized discs consumed by *S. littoralis* larvae during the 8-day experiment; the larvae fed on discs with fungal colonization consuming a mean area of 13.6 ± 0.3 mm^2^ of leaf disc per capita and day and the larvae fed on control discs consuming a mean area of 14.6 ± 0.3 mm^2^ of leaf disc per capita and day. In general, more of the control discs than the *Metarhizium*-colonized discs were consumed daily, except on the second day, when more of the *Metarhizium*-colonized discs than the control discs were consumed (Figure 3). The disc consumption per capita in both treatments was reduced during days 6 to 8, but significant differences were found between the treatments (Figure 3).

#### 3.1.2. Sublethal Reproductive Effects of Exposure of *S. littoralis* to *Metarhizium*-Colonized Melon Discs

The ingestion of *Metarhizium*-colonized discs had a significant effect on the fecundity (χ^2^(1) = 161.95, *p* < 0.001) and fertility (χ^2^(1) = 242.28, *p* < 0.001) of the females that emerged from the surviving pupae developing from larvae feeding on treated melon discs (n_control_ = 26 females, n_colonized_ = 16 females). The highest mean value of fecundity was 221.3 ± 75.0 eggs per female emerging from surviving pupae developing from larvae feeding on control melon discs, while the lowest fecundity value of 135.6 ± 50.3 eggs per female was found in the females emerging from pupae developing from larvae fed on fungus-colonized melon discs. The lowest mean egg fertility (as measured by emerged larvae) was 19.9% for adults emerging from the fungal treatment, compared to 25.8% of egg fertility for the control treatment.

#### 3.1.3. Stress Induced via Caspase Activation Following the Ingestion of *Metarhizium*-Colonized Melon Discs

Dead larvae identified following the ingestion of either *Metarhizium*-colonized of control discs did not show activity of caspases 1, 3/7, and 8, with means ranging from 17.1 to 19.0 RLU for larvae fed on *Metarhizium*-colonized discs and means between 17.7 and 19.2 RLU for larvae fed on control discs.

### 3.2. Effect of in Planta Exposure of S. littoralis to Metarhizium-Colonized Melon Leaves

#### 3.2.1. Mortality and Food Consumption

The percentage of fungal colonization on the *Metarhizium*-treated leaves was 95.0 ± 3.4% at 48 h, while the percentage of fungal colonization on the non-treated leaves from *Metarhizium*-treated plants was 5.8 ± 1.8% (leaves distant from the inoculated ones). The control leaves did not show any signs of fungal colonization. Significant differences in mortality were found between treatments (χ^2^(3) = 27.2, *p* < 0.001), with mortality rates of 12.5 and 70.8% for larvae fed on non-inoculated non-damaged plants versus non-inoculated damaged plants, respectively. However, the mortality rates of the larvae fed on *Metarhizium*-colonized plants were 58.3% and 87.5% for non-damaged and damaged plants, respectively, and Abbott mortalities of 45.8% and 16.7%, respectively (Table 1). The average survival times also showed significant differences, ranging from 3.1 days for *Metarhizium*-colonized damaged plants to 6.9 days for the control and non-damaged plants (Table 1). In addition, the cumulative survival ratio significantly decreased in larvae fed on both *Metarhizium*-colonized and damaged plants (Figure 2B).

Neither *M. brunneum* colonization nor leaf damage had a significant impact on plant consumption (H = 5.44, *p* = 0.1422), with the leaf area consumed by *S. littoralis* larvae of 19.7 ± 5.0 mm^2^ and 24.7 ± 4.6 mm^2^ for non-damaged and damaged control plants, respectively, and 15.1 ± 1.9 mm^2^ and 12.4 ± 2.9 mm^2^ for non-damaged and damaged *Metarhizium*-colonized plants, respectively.

#### 3.2.2. Stress Induced via Caspase Activation after the Exposure of *S. littoralis* to *Metarhizium*-Colonized Melon Leaves

Caspase 1 activity was not detected in larvae that died after exposure to either *Metarhizium*-colonized, non-damaged plants or non-colonized, damaged plant, with means ranging from 18.5 RLU for larvae fed on the controls to 18.8 RLU for larvae fed on *Metarhizium*-colonized, non-damaged plants.

## 4. Discussion

Several studies have evaluated the mortality of *S. littoralis* larvae after the ingestion of EPF-colonized plants [3,17,30]. However, this is the first attempt to elucidate the causes of this mortality and the effects of exposure to EPF-colonized plants both using leaf discs from colonized plants and *in planta*. In both scenarios, leaves treated with the *M. brunneum* EAMb 09/01-Su strain were endophytically colonized at 48 h, at rates of 64.4% for sprayed leaves used to obtain leaf discs and 95.0% for brushed leaves used *in planta*. These values are slightly higher than those reported by Resquín-Romero et al. [3] following the spraying of leaf surfaces of melon plants with the same strain (approximately 50.0% at 48 h) and by Garrido-Jurado et al. [2] after brushing the fungus on melon leaves (approximately 70.0% at 48 h), indicating good experimental conditions for endophytic colonization in which the strain expressed its high endophytic potential. In addition, the *in planta* experiment showed that this strain was able to enter the plant and colonize non-treated areas (5.8% colonization), as previously reported after 96 h of treatment Garrido-Jurado et al. [2].

The mortality of *S. littoralis* larvae fed on *Metarhizium*-colonized discs was similar to that previously reported for the same strain [3], but in this case, the AST was slightly lower (6.6 days) than in the previous study (8.4 days), probably because of the higher colonization rates. Previous studies have shown variability in lepidopteran mortality after the ingestion of EPF-colonized plants. Some of these studies have reported high mortality values after the ingestion of colonized discs [25,31,32], but others have only shown a reduction in fitness [33,34]. In the current study, the fitness of *S. littoralis* larvae feeding on *Metarhizium*-colonized discs and adults emerging from pupae developing from surviving larvae was reduced in terms of food consumption and reproductive potential, respectively. This study showed that *Metarhizium*-colonized discs negatively affected feeding on melon plants. Similarly, McGee [33] and Russo et al. [31,35] reported a reduction in the consumed area of fungal-colonized plants by the noctuids *Helicoverpa armigera* (Hübner), *H. gelotopoeon* (Dyar) and *Rachiplusia nu* (Guenée). However, Viana et al. [36] and Allegrucci et al. [37] reported that noctuid larvae of *H. gelotopoeon* and gelechiid larvae of *Tuta absoluta* (Meyrick) preferred to feed on EPF-colonized discs.

Both the fecundity and fertility of females that emerged from surviving pupae that developed from larvae fed on *Metarhizium*-colonized discs were reduced compared to those of females that emerged from surviving pupae of larvae fed on control discs. In addition, the data obtained in the current work suggest low fertility of eggs laid by *S. littoralis* larvae fed on melon plants. The fecundity and fertility of *S. littoralis* females may be influenced by the host plant; in fact, cucurbitaceans are preferred plants for *S. littoralis* feeding but negatively affect insect reproductive parameters [38,39]. Our results were consistent with previous studies on lepidopteran pests showing a reduction in the lifespan and reproductive potential of insects fed on EPF-colonized plants or their offspring [25,31,36]. These studies also indicate that these effects could be produced as a result of secondary metabolite production or the induction of a systemic response in the colonized plants [31]. Resquín-Romero et al. [3] quantified destruxin A metabolite production on *S. littoralis* cadavers after the ingestion of *Metarhizium*-colonized discs, but the percentage of cadavers with destruxin A (11%) could not entirely explain the lack of fungal outgrowth on dead larvae. Other studies have provided evidence of cellular death or apoptosis in the cadavers of insects fed on endophytically colonized plants [2] or insects that ingested *Metarhizium* conidia, inducing high expression levels of stress genes such as thiol peroxidase and heat shock proteins (HSPs), probably regulating caspase activation and subsequent insect death [22,23,24]. Notably, our results showed for the first time that the cellular death induced in *S. littoralis* cadavers was not caspase-mediated, as observed in aquatic insects such as mosquito larvae and the crustaceans *D. pulex* and *A. magna*. Other studies have revealed that apoptosis in insects may be induced by ribosome-inactivating proteins (RIPs) produced by plants against pathogens and insect pest, and RIPs have been demonstrated to show insecticidal activity and cause fitness reduction in several lepidopteran species [40].

In tritrophic insect–fungus–plant systems, both insects and fungi may induce several plant responses. Differences in the plant volatile profile and secretion of metabolites have been described after insect feeding and fungal infection, as has systemically-acquired resistance, herbivore-induced resistance and induced systemic resistance of plants, with the accumulation of jasmonate and salicylic acid and the activation of resistance genes [41,42]. These processes can be amplified when the host plant is a suboptimal food resource for the insect pest, as observed in our work, in which melon plants were clearly non-favorable for *S. littoralis* in terms of certain parameters of its life table, such as egg fertility [38,39]. In this context, Jaber and Vidal [25] suggested that the loss of volatiles or secretion of metabolites may be induced after mechanical damage to a plant (i.e., the collection of foliar discs). In our study, larval mortality in the *in planta* experiment was higher than that in the experiment with discs on both damaged and *Metarhizium*-colonized plants, probably due to the secretion of compounds such as phenols or saponins, which are known to be toxic to herbivores [43,44,45], and the alteration of hormone signaling after the induction of damage [17,46]. Similar results were obtained with third-instar *S. littoralis* larvae fed on young cotton leaves from damaged and control plants, in which the larvae fed on damaged plants died by day 7 (87.5% mortality), and those fed on control plants pupated successfully, probably due to a reduction in palatability after foliar damage [47,48]. However, the area consumed by the *S. littoralis* larvae on damaged and *Metarhizium*-colonized plants was approximately the same. In this respect, there is no consensus about the potential of damaged or inoculated plants to act as a feeding deterrent [25,49]. This might be due to the few tritrophic studies performed thus far.

## 5. Conclusions

The present work reveals the occurrence of intermediate larval mortality levels related to the foliar application of the entomopathogenic endophytic fungus *M. brunneum* that were not associated with fungal outgrowth and were not caspase mediated. This work also shows the existence of significant sublethal effects of food consumption by larvae challenged by the fungal endophyte when feeding on colonized leaves and effects on the reproduction (female fecundity and egg fertility) of the adults emerging from pupae developing from surviving larvae. Experiments performed with foliar discs provide a biased analysis of this scenario due to the prior damage caused to the plant. The data from this research should be supplemented by the measurement of secreted metabolites, emitted volatiles and the regulation of plant hormones, and the reaction of the immune system of the insects after the ingestion of colonized plants under a scenario involving whole plants.

## Figures and Tables

**Table 1:** Effects of exposure of *S. littoralis* larvae to *M. brunneum*-colonized plants.

	Disc Assay ^c^		*In Planta* Assay ^d^			
			Normal Plant		Damaged Plant	
	Mortality ^a^ (%)	AST ^b^ (days)	Mortality ^a^ (%)	AST ^b^ (days)	Mortality ^a^ (%)	AST ^b^ (days)
Control	11.7 ± 4.2 ^a^	7.5 ± 0.2 ^a^	12.5 ± 12.5 ^a^	6.9 ± 0.7 ^a^	70.8 ± 7.7 ^b^	3.3 ± 0.6 ^a^
Colonized	45.0 ± 1.4 ^b^	6.6 ± 0.2 ^b^	58.3 ± 12.4 ^b^	5.2 ± 0.6 ^a^	87.5 ± 8.5 ^b^	3.1 ± 0.5 ^a^

^a^ Within the same column, means with the same letter are not significantly different to each other (χ^2^ test, *p* ≤ 0.05) according to the generalized linear model. Data are expressed as mean ± SE. ^b^ AST: Average survival time was limited to 8 days. Within the same column, means with the same letter are not significantly different to each other (*p* ≤ 0.05) according to the log-rank test. Data are expressed as mean ± SE. ^c^ n_control_ = 120 and n_colonized_ = 120. ^d^ n_control/non-damaged_ = 24, n_control/damaged_ = 24, n_colonized/non-damaged_ = 24, n_colonized/damaged_ = 24.
